# Improved genomic prediction of clonal performance in sugarcane by exploiting non-additive genetic effects

**DOI:** 10.1007/s00122-021-03822-1

**Published:** 2021-04-26

**Authors:** Seema Yadav, Xianming Wei, Priya Joyce, Felicity Atkin, Emily Deomano, Yue Sun, Loan T. Nguyen, Elizabeth M. Ross, Tony Cavallaro, Karen S. Aitken, Ben J. Hayes, Kai P. Voss-Fels

**Affiliations:** 1 Queensland Alliance for Agriculture and Food Innovation, Queensland Bioscience Precinct, Carmody Rd., St. Lucia, Brisbane, QLD 3064067 Australia; 2 Sugar Research Australia, Mackay, QLD 4741 Australia; 3 Sugar Research Australia, 50 Meiers Road, Indooroopilly, QLD 4068 Australia; 4 Sugar Research Australia, Meringa, Gordonvale, QLD 4865 Australia; 5 Agriculture and Food, CSIRO, QBP, St. Lucia, QLD 4067 Australia

## Abstract

**Key message:**

Non-additive genetic effects seem to play a substantial role in the expression of complex traits in sugarcane. Including non-additive effects in genomic prediction models significantly improves the prediction accuracy of clonal performance.

**Abstract:**

In the recent decade, genetic progress has been slow in sugarcane. One reason might be that non-additive genetic effects contribute substantially to complex traits. Dense marker information provides the opportunity to exploit non-additive effects in genomic prediction. In this study, a series of genomic best linear unbiased prediction (GBLUP) models that account for additive and non-additive effects were assessed to improve the accuracy of clonal prediction. The reproducible kernel Hilbert space model, which captures non-additive genetic effects, was also tested. The models were compared using 3,006 genotyped elite clones measured for cane per hectare (TCH), commercial cane sugar (CCS), and Fibre content. Three forward prediction scenarios were considered to investigate the robustness of genomic prediction. By using a pseudo-diploid parameterization, we found significant non-additive effects that accounted for almost two-thirds of the total genetic variance for TCH. Average heterozygosity also had a major impact on TCH, indicating that directional dominance may be an important source of phenotypic variation for this trait. The extended-GBLUP model improved the prediction accuracies by at least 17% for TCH, but no improvement was observed for CCS and Fibre. Our results imply that non-additive genetic variance is important for complex traits in sugarcane, although further work is required to better understand the variance component partitioning in a highly polyploid context. Genomics-based breeding will likely benefit from exploiting non-additive genetic effects, especially in designing crossing schemes. These findings can help to improve clonal prediction, enabling a more accurate identification of variety candidates for the sugarcane industry.

## Introduction

Modern sugarcane cultivars are highly heterozygous due to their interspecific origin and high polyploidy (Garsmeur et al. 2018; Piperidis et al. 2010). Genetic improvement by selection has substantially contributed to the cane and sugar yield of modern cultivars (Ming et al. 2010). However, over the past two decades, the genetic improvement rate has been low or static in sugarcane compared to other major crops (Wei and Jackson 2016; Yadav et al. 2020).

In the past decade, animal and plant breeding has been revolutionized by genomic selection (GS) (Meuwissen et al. 2001), which is now widely implemented as a powerful breeding tool in modern breeding programs (Heffner et al. 2009; Jannink et al. 2010). GS offers new opportunities for accurate prediction of non-phenotyped clones in sugarcane, which could help breeders to accelerate the improvement of complex traits (Deomano et al. 2020; Gouy et al. 2013; Hayes et al. 2020).

The objective of most breeding programs is to maximize the average performance of future generations by selecting and combining genetically superior individuals in the current generation. Genetically superior individuals as parents are typically selected based on their breeding values (BVs), which are assumed to be additive and capture a substantial amount of the genetic variation for a trait of interest. Because it is the additive component that is passed on from the parents to the offspring, most genomic evaluation models use an underlying additive genetic model to estimate allele substitution effects either explicitly by estimating the effect of each marker or implicitly through the genomic relationship matrix using genome-wide DNA markers (Meuwissen et al. 2001; VanRaden 2008).

Several factors determine genomic prediction accuracies, such as training population size, the heritability of the trait of interest, marker density, the statistical model, and the genotyping platforms used (Heffner et al. 2009; Heslot et al. 2012). However, one of the key factors of genomic prediction accuracy is the genetic architecture of the trait, particularly how many loci and which gene action (additive/non-additive) control the trait (Daetwyler et al. 2010; Toro and Varona 2010). Most agronomically and economically important traits in sugarcane, such as tonnes cane per hectare (TCH) and sugar content determined as commercial cane sugar (CCS), are highly quantitative and complex and assumed to be controlled by many loci (Casu et al. 2005; Hoang et al. 2015).

For commercial sugarcane breeding where highly heterozygous clones are vegetatively propagated, the performance of the progeny is determined by both additive contributions of the parents and interactions of alleles within loci (dominance) and among loci (epistasis). It has been widely reported in the literature that variation for cane yield can substantially be affected by non-additive genetic effects, which is also reflected by low narrow-sense heritability estimates for TCH (Hogarth 1971; Hogarth et al. 1981; Jackson and McRae 2001; Pisaroglo De Carvalho et al. 2014). Since one of sugarcane breeding's main objectives is to develop new varieties with improved overall performance as determined by both additive and non-additive genetic components, breeding approaches that focus only on increasing the breeding value might not be adequate. Therefore, approaches that also target non-additive genetic effects should be considered.

The estimation of non-additive genetic effects is statistically and computationally challenging, which is also one reason why they are often not considered in genetic evaluation models (Varona et al. 2018). However, if traits are under substantial non-additive genetic control, a genomic model with only additive effects can lead to biased breeding value estimates (e.g. Hunt et al. (2020). The consideration of non-additive genetic effects in genetic evaluations can potentially improve the accuracy of prediction of breeding values and clonal performance (total genetic value) and, therefore, have a direct positive impact on the response to selection in a breeding program.

The availability of high-density genome-wide SNP markers has made it feasible to investigate the role of non-additive genetic effects for complex traits using statistical genomic evaluation models, most of which were developed for diploid species. Despite the advancements in sequencing and genotyping technologies, extreme levels of ploidy remain a challenge for genomic studies in many polyploid species. In the highly polyploid crop sugarcane, single-dose markers (SDMs) have become the primary choice for linkage analysis because their segregation pattern follows that of diploid species (1:1 or 3:1 in bi-parental population), even though the exact level of ploidy and allele dosages are uncertain (Aitken et al. 2016; Wu et al. 1992). This implies that under these circumstances, using statistical and genetic tools that were developed for diploids can give an appropriate approximation of the underlying complexity, while there doubtlessly is need for further work that enables to better deal with the extreme levels of genomic complexity in sugarcane.

From a computational point of view, the most efficient way for including non-additive genetic effects in genetic evaluation models is to define appropriate covariance matrices between individual effects, in the same way that the standard GBLUP model uses the additive genomic relationship matrix (Vitezica et al. 2017, 2013). Semi-parametric reproducing kernel Hilbert space (RKHS) regression models have also been advocated as a potential alternative to capture non-additive effects in genomic selection (Gianola et al. 2006; Gianola and van Kaam 2008). The RKHS approach incorporates the kernel matrix, which is constructed by the use of squared Euclidean distances between markers to quantify the degree of relatedness between individuals. It was demonstrated that if an appropriate kernel is used (e.g. Gaussian kernel), the RKHS model also captures epistasis (especially additive-by-additive) effects (Jiang and Reif 2015).

The decomposition of genetic variance of complex traits into additive and dominance variance has been reported in several genomic prediction studies (Aliloo et al. 2016; Hunt et al. 2020; Su et al. 2012). Some studies have also attempted to model epistasis by including additive–additive, additive–dominant, and dominant–dominant second-order epistatic interaction effects between SNP, e.g. in hybrid wheat (Boeven et al. 2020), Nile tilapia (Joshi et al. 2020), and tropical beef cattle (Raidan et al. 2018). For improving production traits in dairy cattle, the explicit consideration of dominance genetic effects in genomics-based mating scheme designs was advantageous over approaches that only considered additive genetic effects (Aliloo et al. 2016, 2017; Jiang et al. 2017). An increase in genomic prediction accuracy for multi-environment-drought tolerance in maize has been reported when additive and dominance effects were included in the statistical model (Dias et al. 2018). In a multi-environment sorghum study, the authors concluded that incorporating dominance genetic effects together with additive effects could help to more accurately predict hybrid performance in distinct environments (Hunt et al. 2020). A substantial amount of non-additive genetic variance has also been reported for important traits in autopolyploid potato, such as yield, specific gravity, frycolour, and specific consideration of non-additive effects in genomic prediction models improved the prediction accuracy of total genotypic values (Endelman et al. 2018).

However, in some genomic studies on crossbred species, it was reported that non-additive genetic effects (especially dominance) contributed significantly to the total genetic variance, but no improvement in predictive ability was noticed when including these effects in the genomic prediction model (Moghaddar and van der Werf 2017; Xiang et al. 2016). In a recent study, Amadeu et al. (2020) compared a polyploid vs. diploid parameterization to account for allele dosage in genomic prediction in the autotetraploids potato and blueberry and found that the diploid parameterization gave similar results like the polyploid parameterization for important agronomic traits such as yield. They suggested that the pseudo-diploid parameterization could in theory be applied to any level of ploidy, although the literature on specific dominance interaction estimations for higher ploidy levels is not yet available. Matias et al. (2019) reported similar results for the autotetraploid *Urochloa spp*. It is therefore unclear whether a pseudo-diploid model can provide an appropriate approximation for extreme levels of ploidy like in sugarcane, and if the inclusion of non-additive genetic effects in genomic prediction models can improve the prediction accuracy of breeding values and clonal performance in sugarcane.

Genomic prediction is relatively new in sugarcane, and there are only a few studies reported the accuracy of important economic traits. The first genomic prediction study on sugarcane was described by Gouy et al. (2013), and further research is ongoing to assess the full potential of GS for complex trait improvement. Gouy et al. (2013) showed promising results in relatively small elite populations which was confirmed by recent studies that investigated  larger commercial populations (Deomano et al. 2020; Hayes et al. 2020). Most of these studies have modelled additive effects, while Deomano et al. (2020) also tried RKHS with a Gaussian kernel, which captures non-additive effects to predict the clonal performance of untested clones. However, none of these studies has attempted to specifically account for non-additive genetic effects in both variance partitioning and genomic prediction of clonal performance.

Therefore, the key objectives of this study were to i) decompose the total genetic variance into additive and non-additive genetic variance using a diploid parameterization based on genome-wide SNP that were largely reported to be single dose (as an approximation for the extreme ploidy in sugarcane), for the three economically important traits TCH, CCS, and Fibre content and ii) to investigate whether including non-additive genetic effects and overall genome-wide heterozygosity improves the prediction accuracy of clonal performance. To address these objectives, we used a large data set from a commercial sugarcane breeding program comprising 3,006 clones genotyped with 26,000 SNP markers that were phenotyped in multi-environment field trials in four major growing regions across five years. We provide variance component estimates for key traits and compare genomic prediction accuracies obtained from fitting a conventional GBLUP model, extended-GBLUP models that specifically include dominance, epistatic, and genome-wide heterozygosity effects, and an RKHS model using a Gaussian kernel in two forward prediction scenarios.

## Materials and methods

### Reference population and genotyping

A total of 3,006 clones were genotyped using a 70 K SC_Affymetrix Axiom cane SNP array (Aitken et al. 2016). The array contained 58,028 single-dose polymorphic SNPs and was developed using Australian and Brazilian germplasm for sugarcane breeding programs. A pseudo-diploid model of genotype calling was used where all heterozygous genotypes were considered as one genotypic class, which is similar to the genotype calling procedures used in previous GS studies in sugarcane (Deomano et al. 2020; Hayes et al. 2020). SNP data were coded as 0 and 2 for homozygous for the reference and alternate allele, respectively, and 1 for the heterozygous genotype. More information about the array and genotype calling can be found in Aitken et al. (2016). The marker data were pre-processed using the R-package “SelectionTools” version 19.3. Low-frequency markers (terminology adopted from Gouy et al. (2013)), i.e. SNP for which the frequency of the alternate allele was less than 0.01, were removed. The minimum individual and locus call rate was set to 90%, respectively. After filtering, we ended up with a total of 25,753 high-quality SNPs and 2,909 clones that were tested in evaluation trials between 2013 and 2017.

### Phenotype data and statistical analyses

The population used in this study consisted of final assessment trial (FAT) clones. These clones were obtained by intensive previous selection from two earlier stages, i.e. progeny assessment trials (PATs) and clonal assessment trials (CATs). The initial population was established with 25,000 genotypes from about 250–300 bi-parental crosses at each of the four selection programs in Australia. Due to a general breeding selection bottleneck in sugarcane and also a limited number of opportunities for chromosome recombination from the original founders (only 7–9 breeding cycles in modern breeding so far), most of the parents are related and a reasonable amount of linkage disequilibrium exists in Sugar Research Australia’s (SRA) breeding populations. In addition, population structure has been found in SRA’s breeding populations although an exact classification of subpopulations is not clear. FATs used in this study were established in the sugarcane breeding programs of SRA in Queensland’s four growing regions in “Northern,” “Burdekin,” “Central,” and “Southern” with four trials  per region and per year from 2013 to 2017. All trials planted in one year are referred to as a series, as each trial is harvested three times in three years. After planting, the first harvest is named as plant crop, the following two harvests as first and second ratoon crops. Clones at each series were mostly repeated over at least three locations within a region but generally uncommon across regions, except for a few common standards. A partially replicated design with four rows by 10-m plots was used in each trial, with an average replication of 22% within each trial. The data for TCH were collected from the middle two-row plots, and the measurement of CCS and Fibre content was mostly done by using the SpectraCane™ (Berding and Marston 2010) or press method (BSES 2001) with six randomly selected stalks. Data were collected from three crops: plant, first, and second ratoon crops. More information on data collection is available in Deomano et al. (2020).

The genomic predictions were performed using a two-stage approach. First, to account for spatial variation in each trial and genotype-by-environment interaction (GEI) between trials and crops, a linear mixed model was fitted by considering the spatial and GEI effects to estimate each clone's best linear unbiased prediction (BLUP) within a year-crop using the BLUP methodology described in Smith et al. (2007). SRA provided these BLUPs corrected for spatial effects for 3,006 clones from 2013 to 2017 FATs for TCH, CCS, and Fibre content. In the first step, no pedigree information was fitted to obtain the BLUPs which means there was no “shrinkage” of the phenotypes towards the pedigree before the genomic prediction models were fitted. Best linear unbiased estimates (BLUEs) are used as phenotypes in genomic prediction in most studies; however, BLUPs were available through the commercial partner which helped to accommodate unbalancedness and a small extent of missing information in the data set. In the second step, breeding values or total genetic values were computed using genomic covariance matrices and adjusted phenotypes from the first stage as response variables. For traditional GBLUP (purely additive) model, breeding values are obtained as GEBVs; however, the total genetic value of clones is obtained through genomic prediction of clonal performance (GPCP), which is estimated using the extended-GBLUP and RKHS models and therefore includes additive and non-additive genetic effects. The prediction accuracy of clones in validation years was compared to GBLUP, extended-GBLUP (including non-additive genetic effects and heterozygosity), and RKHS, a non-parametric approach which can capture epistatic effects (Jiang and Reif 2015).

### Construction of genomic relationship matrices (GRM)

The additive ($G_{A}$), dominance ($G_{D}$), and additive–additive epistatic (*G*_AA_) relationship matrices were computed by adopting the natural and orthogonal interactions (NOIA) approach proposed by Alvarez-Castro and Carlborg (2007). In this approach, incidence matrices are built based on genotypic frequencies without the assumption of Hardy–Weinberg equilibrium (HWE) (Alvarez-Castro and Carlborg 2007; Vitezica et al. 2017).

### Additive genomic relationship matrix ($G_{A}$)

The additive genomic relationship matrix was constructed as $G_{A} = \frac{{H_{A} H_{A}^{^{\prime}} }}{{{\text{tr}}\left( {H_{A} H_{A}^{^{\prime}} } \right)/n}}$, where $H_{A}$ is the incidence matrix of additive genetic effects, *tr* is the trace of the matrix, and *n* denotes the total number of individuals. The dimension of the matrix $H_{A }$ is the number of individuals *n* (rows) by *p* number of markers (columns). $H_{A}$ was obtained as $H_{A}$ = $$\left\{ { \begin{array}{*{20}c} { h_{Ak} } \\ . \\ . \\ {h_{An} } \\ \end{array} } \right.$$, where each $h_{{{\text{Ak}}}}$ represents a row vector for the *k*_th_ individual with *p* (number of markers) columns. The elements of $h_{Ak}$ were obtained as.

$$h_{A} = \left\{ {\begin{array}{*{20}c} { - \left( { - p_{{{\text{Aa}}}} - 2p_{{{\text{aa}}}} } \right)} \\ { - \left( {1 - p_{{{\text{Aa}}}} - 2p_{aa} } \right)} \\ { - \left( {2 - p_{{{\text{Aa}}}} - 2p_{{{\text{aa}}}} } \right)} \\ \end{array} } \right.$$ for genotypes $$\left\{ {{ }\begin{array}{*{20}c} {{\text{AA}}} \\ {{\text{Aa}}} \\ {{\text{aa}}} \\ \end{array} } \right.$$ where $p_{{{\text{aa}}}}$, $p_{{{\text{Aa}}}}$ represent the genotypic frequencies of aa and Aa in the current population.

### Dominance genomic relationship matrix ($G_{D}$)

The dominance genomic relationship matrix was constructed as $G_{D} = \frac{{H_{D} H_{D}^{^{\prime}} }}{{{\text{tr}}\left( {H_{D} H_{D}^{^{\prime}} } \right)/n}}$, where $H_{D}$ is the incidence matrix of dominance genetic effects. Similarly, the dimension of $H_{D}$ is *n* (number of individuals) by *p* (number of markers). $H_{D}$ was obtained as $H_{D}$ = $$\left\{ { \begin{array}{*{20}c} { h_{{{\text{Dk}}}} } \\ . \\ . \\ {h_{{{\text{Dn}}}} } \\ \end{array} } \right.$$, where each $h_{{{\text{Dk}}}}$ represents a row vector of length p (number of markers) for the *k*th individual.

$$h_{D} = \left\{ {\begin{array}{*{20}c} {\frac{{ - 2p_{{{\text{Aa}}}} p_{{{\text{aa}}}} }}{{p_{{{\text{AA}}}} + p_{{{\text{aa}}}} - \left( {p_{{{\text{AA}}}} - p_{{{\text{aa}}}} } \right)2}}} \\ {\frac{{4p_{{{\text{AA}}}} p_{{{\text{aa}}}} }}{{p_{{{\text{AA}}}} + p_{{{\text{aa}}}} - \left( {p_{{{\text{AA}}}} - p_{{{\text{aa}}}} } \right)2}}} \\ {\frac{{ - 2p_{{{\text{AA}}}} p_{{{\text{Aa}}}} }}{{p_{{{\text{AA}}}} + p_{{{\text{aa}}}} - \left( {p_{{{\text{AA}}}} - p_{{{\text{aa}}}} } \right)2}}} \\ \end{array} } \right.$$ for genotypes $$\left\{ { \begin{array}{*{20}c} {{\text{AA}}} \\ {{\text{Aa}}} \\ {{\text{aa}}} \\ \end{array} } \right.$$

The genotypic frequencies of the genotypes AA, Aa, and aa are represented by $p_{AA} ,p_{Aa} , p_{aa}$, respectively.

### Epistatic genomic relationship matrices (*G*_AA,_*G*_AD,_*G*_DD_)

The second-order epistatic relationship matrices (additive–additive, additive–dominance, or dominance–dominance) were  built by calculating Hadamard products (symbolized as $$\odot$$) of the respective covariance matrices. The additive–additive relationship matrix is computed as the Hadamard product of the additive ($$G_{A} )$$ matrix, i.e. as $$G_{{{\text{AA}}}} = \frac{{G_{A} \odot G_{A} }}{{{\text{tr}}\left( {G_{A} \odot G_{A} } \right)/n}}$$. Similarly, the additive–dominance and dominance–dominance relationship matrices were calculated as $$G_{{{\text{AD}}}} = \frac{{G_{A} \odot G_{D} }}{{tr\left( {G_{A} \odot G_{D} } \right)/n}}$$ $$G_{{{\text{DD}}}} = \frac{{G_{D} \odot G_{D} }}{{{\text{tr}}\left( {G_{D} \odot G_{D} } \right)/n}}$$, respectively. This approach can be extended easily to third and high order interactions (Vitezica et al. 2017). All relationship matrices were standardized by dividing by the average of the main diagonal.

### Average genome-wide heterozygosity

For each clone, the average genome-wide heterozygosity was calculated directly from the dominance covariate matrix, $H_{D}$ following the approach by Aliloo et al. (2017) as

$${\text{Het}}_{k} = \frac{{\mathop \sum \nolimits_{l = 1}^{p} h_{kl} }}{{\mathop \sum \nolimits_{l = 1}^{p} 2p_{l} q_{l} }}$$

where $Het_{k}$ is the average genome-wide heterozygosity of individual *k* averaged across all SNP markers and $h_{kl}$ is the corresponding element of $H_{D}$ for individual *k* at the *l*th SNP; *p*_*l*_ and *q*_*l*_ are the frequency of the reference and the alternate allele at *l*_*th*_ SNP.

### Genomic prediction models

#### GBLUP and extended-GBLUP

Variance components were estimated using six univariate GBLUP models. The base additive model, including random additive genetic effects (A), was extended to a model with random additive and dominance genetic effects (AD) and gradually extended to a full model with random additive, dominance, and additive–additive epistatic interaction effects (ADE). All these genomic models (A, AD, ADE) were further expanded by incorporating the average genome-wide heterozygosity as a fixed regression coefficient, resulting in the following six models:

i. Model (**A**): $$y^{n \times 1} = X\beta + {\text{Wa}} + \varepsilon$$
ii. Model (AH): $y^{n \times 1} = X\beta + {\text{Wa}} + b\left( {{\text{Het}} - \overline{{{\text{Het}}}} } \right) + \varepsilon$
iii. Model (AD): $y^{n \times 1} = X\beta + {\text{Wa}} + {\text{Wd}} + \varepsilon$
iv. Model (ADH): $y^{n \times 1} = X\beta + {\text{Wa}} + {\text{Wd}} + b\left( {{\text{Het}} - \overline{{{\text{Het}}}} } \right) + \varepsilon$
v. Model (ADE): $$y^{n \times 1} = X\beta + {\text{Wa}} + {\text{Wd}} + {\text{Wt}} + \varepsilon$$
vi. Model (ADEH): $y^{n \times 1} = X\beta + {\text{Wa}} + {\text{Wd}} + {\text{Wt}} + b\left( {{\text{Het}} - \overline{{{\text{Het}}}} } \right) + \varepsilon$

In general, the model *Y*_BLUPs_ = mean + Year + Crop + Region + Trial + Clone + residual was fitted for all traits (TCH, CCS, and Fibre) for the second-stage analysis. Each model term except for the clone was treated as a fixed effect. Because the data set was unbalanced with only a small fraction of clones tested across regions and years (between 5 and 20 clones, depending on the region-year combination), we did not include interaction terms in our prediction models.

For all models, y is the vector of adjusted phenotypes corrected for spatial effects from each trial, $\beta$ is the vector of fixed effects, i.e. the overall mean and all other model terms defined above except clone, and $\varepsilon$ is a vector of the random residual effects following $\sim N(0,$ $I \sigma_{\varepsilon }^{2} )$, where $\sigma_{\varepsilon }^{2}$ is the residual variance. $a$ is the vector of additive genetic effects following $a \sim N(0,$ $G_{A} \sigma_{A}^{2} )$, where $G_{A}$, is the additive genomic relationship matrix and $\sigma_{A}^{2}$ is the additive genetic variance captured by the SNP markers. The incidences matrix $X$ and $W$ is relating observations in y to fixed and random effects. The random dominance effect is defined by *d* following $\sim N(0,$ $G_{D} \sigma_{D}^{2} )$, where $G_{D}$ is the dominance relationship matrix as mentioned above and $\sigma_{D}^{2}$ is the corresponding dominance genetic variance. *t* is the vector of the random additive–additive epistatic effects following $t \sim N(0,$ $G_{{{\text{AA}}}} \sigma_{E}^{2} )$ for the genetic variance components analysis using a genomic additive–additive relationship matrix $G_{{{\text{AA}}}}$. *b* represents the linear regression coefficient of average genome-wide heterozygosity (Het) computed from the dominance covariate matrix $H_{D }$, as described above.

For each model, we calculated both narrow-sense (*h*^*2*^) and broad-sense heritabilities (*H*^*2*^), which correspond to the proportion of phenotypic variance explained by additive genetic variance only (*h*^*2*^) or by additive and non-additive genetic variance combined (*H*^*2*^). Narrow-sense heritability was estimated as $h^{2} = \frac{{\sigma_{A}^{2} }}{{\sigma_{P}^{2} }}$ where $\sigma_{A}^{2}$ is the additive genetic variance estimated using SNP markers and $\sigma_{P}^{2}$ represents the phenotypic variance, which is the sum of genetic and the residual variance. Broad-sense heritability for the AD model was estimated as $H^{2} = \frac{{\sigma_{A}^{2 } + \sigma_{D}^{2 } }}{{\sigma_{P}^{2} }}$, where $\sigma_{D}^{2 }$ represents the estimated dominance variance, and $H^{2}$ for the ADE model was estimated as $H^{2} = \frac{{\sigma_{A}^{2 } + \sigma_{D}^{2 } + \sigma_{E}^{2 } }}{{\sigma_{P}^{2} }}$, where $\sigma_{E}^{2 }$, is the estimated additive–additive epistatic variance. All analyses were performed in ASReml-R (Version 4) (Butler. et al. 2009).

### Reproducible Kernel Hilbert space (RKHS)

In addition to the above models, we fitted the RKHS model to predict clonal performance. We considered the Bayesian RKHS regression model, whose structure was equivalent to the standard animal model suggested by De Los Campos et al. (2010).

$y_{BLUPs} = 1\mu + g + \varepsilon$, with $g \sim N (0,K\sigma_{g}^{2}$), and $\varepsilon \sim N (0,I\sigma_{\varepsilon }^{2}$), where K = $\left\{ {K\left( {x_{i} ,x_{j} } \right)} \right\}$, is a positive semi-definite matrix of dim n × n with n being the number of clones. The kernel matrix elements are calculated as the squared Euclidean distance for pairs of SNP markers between two individuals.

In this study, we considered the Gaussian kernel, $K\left( {x_{i} ,x_{j} } \right) = exp\left\{ {\frac{{ - h \mathop \sum \nolimits_{k = 1}^{p} \left( {x_{ik} - x_{jk} } \right)^{2} }}{p}} \right\}$ (Gianola and van Kaam 2008) where $h$ is the bandwidth parameter, and *p* is the total number of markers. The bandwidth parameter $h$ controls the rate of decay of correlation when the distance between the pair of vectors increases. A large Euclidean distance gives a small kernel value that results in a small spatial genetic similarity between two individuals.

**Fig. 1:**
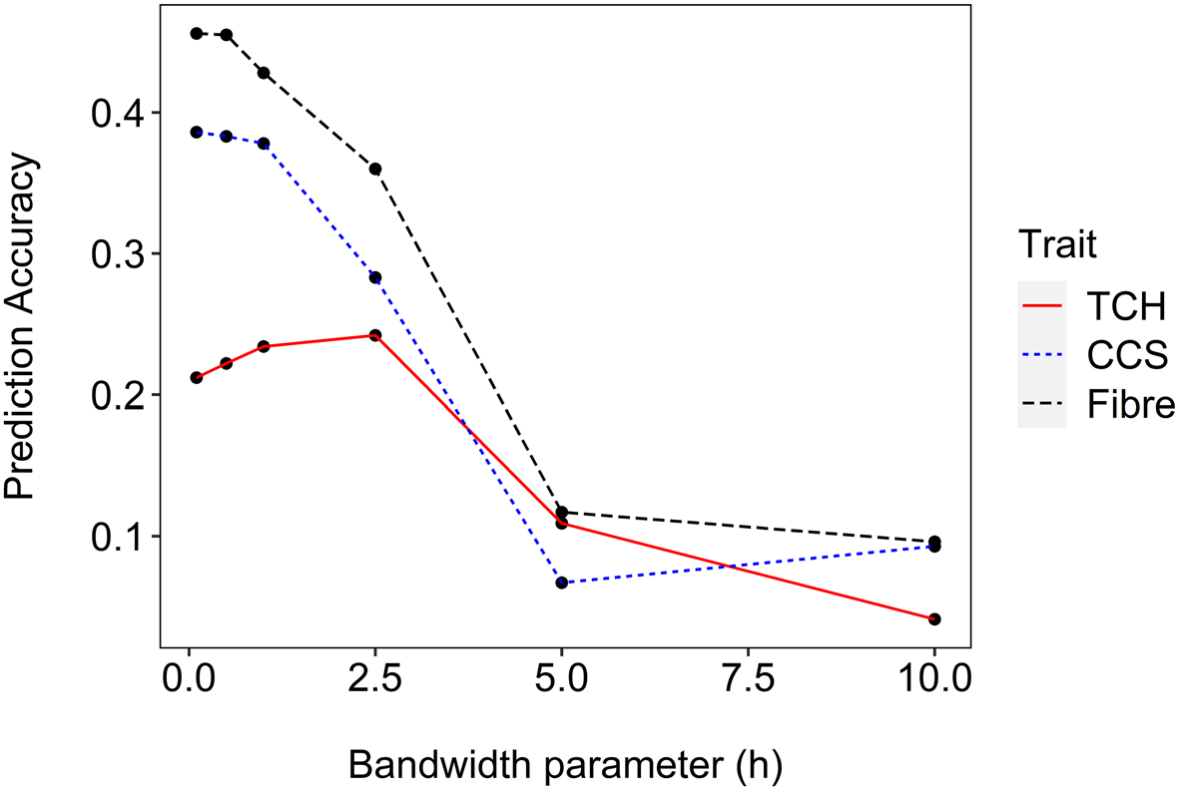
Accuracies of genomic prediction using a single kernel approach in a reproducible kernel Hilbert space (RKHS) model for a range of bandwidth parameters, *h*. This validation of *h* values was performed in order to select *h* values associated the highest prediction accuracy. A validation data set independent of the three forward prediction scenarios was chosen by using 1,320 clones from 2013 and 2014 as training population to predict 662 clones from 2015. TCH = tonnes of cane per hectare; CCS = commercial cane sugar; Fibre = Fibre content

For the Gaussian kernel, the bandwidth parameter *h* can be chosen either using Bayesian or cross-validation techniques. In our study, on the basis of reference values suggested by Perez and de Los Campos (2014), we evaluated a range of *h* = (0.1, 0.5, 1.0, 2.5, 5.0, 10) values to identify which *h* value gives the highest prediction accuracy (Fig. 1). To test the range of potential *h* parameters, we used clones from 2013 to 2014 as the training population and 2015 series clones as the prediction population. Afterwards, a multi-kernel model was fitted with three kernels based on the three *h* values associated with the highest prediction accuracy.

We used the model $y_{{{\text{BLUPs}}}} = 1\mu + \mathop \sum \nolimits_{m = 1}^{3} g_{m} + \varepsilon$, with $g_{m} \sim N (0,K_{m} \sigma_{{g_{m} }}^{2}$), where $K_{m}$ represents the reproducible kernel assessed at the *m*^*th*^ value of the chosen bandwidth parameter *h* = (0.1, 0.5, 1.0). The R-package BGLR was used to fit the RKHS model. Our results were based on a Gibbs sampling process with 5,000 iterations, of which the first 1,000 were discarded as burn-in (Perez and de Los Campos 2014).

### Model comparison

To compare the goodness of fit between the nested GBLUP models, we performed likelihood ratio tests (LRT), using the test statistic = 2 × [max (logL(model AD))—max (logL(model A))], where in this example model A and model AD are nested models with either additive genetic effects (model A) or additive and dominance genetic effects (model AD). The test statistic approximates a chi-squared distribution with one degree of freedom at *p* < 0.05. The model is considered significant if the test statistic > $X_{\left( 1 \right)}^{2}$ at *p* < 0.05.

### Genomic prediction accuracies

Three forward prediction scenarios were considered, to investigate i) the effect of training-population size and ii) the effect of the year-by-year variation on the prediction accuracy for all three traits. In prediction scenarios 1a and 1b, the prediction model was calibrated using 2013–2015 series data as a training set and either 2016 series data (1a, 739 clones) or 2017 series (1b, 691 clones) as a prediction set. Prediction scenario 2 was designed to represent a situation in which the prediction model was updated by including 2016 in the 1a/1b training data set to predict 2017 series clones. Care was taken to ensure there were no overlapping clones in the reference and validation sets. In prediction scenario 1a/b and scenario 2 overlapped clones and SNPs were 739 and 25,714, respectively. The prediction accuracy was calculated as the Pearson’s correlation (*r*) between GEBV (from the additive GBLUP model) or GPCP (from the extended-GBLUP including non-additive effects and RKHS models) and the adjusted phenotypes of those clones in each of the four regions.

## Results

### Estimates of variance components and heritabilities

**Fig. 2:**
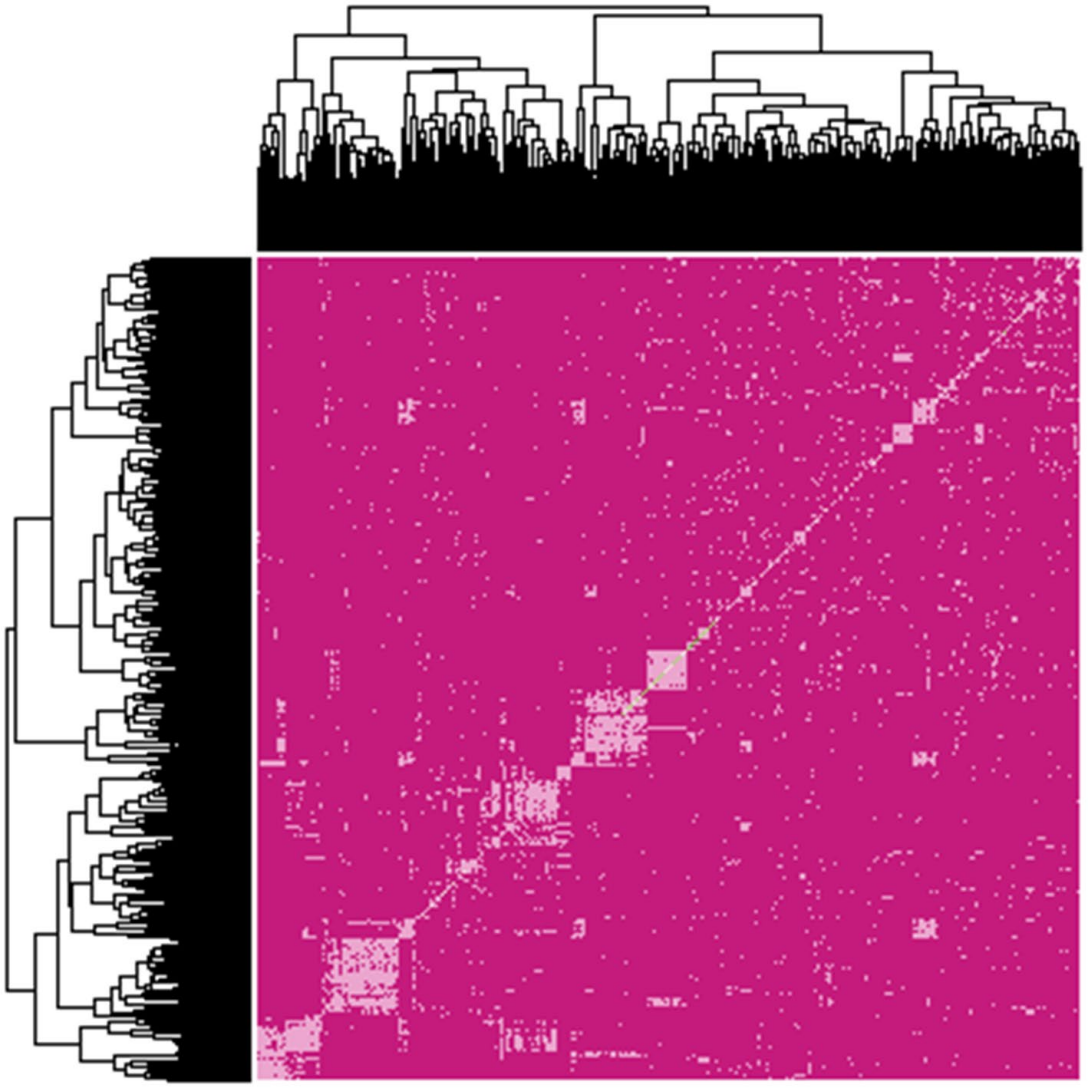
Genomic additive relationship matrix showing the proportion of genome shared amongst a total of 2,909 clones from 2013 to 2017 that were evaluated in final assessment trials of the Sugar Research Australia breeding program. The top and the side axis both represent the clones. Each coloured point represents the proportion of the genome each pair of clones have in common. Higher degrees of genomic relationships between clones are represented by a light colour (e.g. diagonal elements), while a pink shading represents a weaker genomic relationship.

**Fig. 3:**
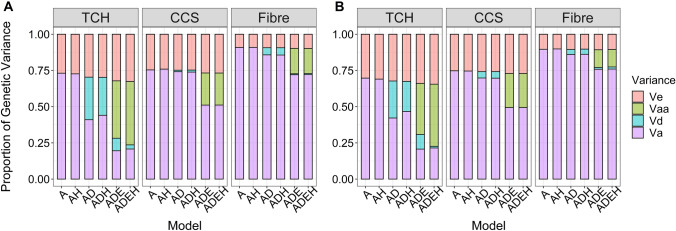
Decomposition of genetic variance into additive, dominance, additive–additive epistatic, and residual variance in two forward prediction scenarios. **a** Proportion of genetic variance in forward prediction scenarios 1 a/1b (1,825 clones from 2013–2015 used as training population) for six different covariance structures (see Table 2). **b** Proportion of genetic variance in forward prediction scenario 2 (2,397 clones from 2013–2016 used as training population) for six different covariance structures (see Table 2). Va = additive genetic variance; Vd = dominance genetic variance; Vaa = additive–additive epistasis variance; Ve = error variance; Model A = additive model; Model *AH* additive plus heterozygosity; Model *AD* additive plus dominance model; Model *ADH* additive, dominance plus heterozygosity; Model *ADE*  additive, dominance and epistatic effect; Model *ADEH*  additive, dominance, epistatic plus heterozygosity; *TCH* tonnes of cane per hectare; *CCS*   commercial cane sugar; Fibre = Fibre content

The genomic relationship matrix ($G_{A}$), revealed a small amount of population structure present, with a few groups of clones highly related to each other (Fig. 2). The estimates for the variance components, heritabilities, genome-wide average heterozygosity effects, and likelihood ratio values obtained from the six different GBLUP models are presented in Tables 2 and 3 for forward prediction scenarios 1a and 1b and scenario2, respectively. The estimates of broad-sense (*H*^*2*^) and narrow-sense heritability (*h*^*2*^) were similar across all evaluated parametric models in all considered scenarios (Table 2 and 3). The estimates of the proportion of the additive variation captured by the SNP markers (SNP heritability) were high among all traits, with the SNP explaining up to 90% of the variation in Fibre. Variance component estimates for the three traits were similar across all three forward prediction scenarios (Tables 2 and 3). The variance component decompositions based on the two training populations for scenarios 1a and 1b and scenario 2 are visualized in Fig. 3.

For TCH, the estimates of additive genetic variance from model A were much larger than from models AD and ADE (Tables 2 and 3), while the residual variances remained constant. It implies that if dominance and epistasis were not fitted in the model, the additive genetic variance component included large portions of non-additive genetic variance. Using model AD for TCH, a large portion of the variance captured by the SNP markers was attributed to dominance (29 and 26% of the total phenotypic variance for scenarios 1 and 2, respectively), and estimates of additive variance were substantially reduced. For TCH, additive–additive interaction explained up to about 58% and 53% of the total genetic variance captured by the SNP markers in scenarios 1 and 2, respectively, while additive variance only explained about 30% in both scenarios (Tables 1 and 2). After including epistatic effects in the model, dominance variance relative to total genetic variance was reduced from 42 to 13% in scenario1 (Fig. 3a) and from 38 to 15% in scenario 2 (Fig. 3b).

**Table 1:** Number of records for Final Assessment Trial (FAT) clones in reference and prediction sets per region

Year	Burdekin	Central	Northern	Southern	Total
2013	322	185	156	155	818
2014	339	139	163	178	819
2015	298	125	156	172	751
2016	320	132	162	196	810
2017	306	187	147	157	797
Total	1585	768	784	858	3995

In forward prediction scenario1a/b 1,825 unique clones from 2013–2015 were used as training set to predict 739 and 691 unique clones from 2016 and 2017, respectively. In forward prediction scenario 2, 2,397 unique clones from 2013–2016 were used as training set to predict 691 unique clones from 2017. Clones that overlapped between the training and prediction sets were removed from the analyses. Overall, 739 clones and 25,714 SNPs overlapped between training populations for prediction scenarios 1a/b and scenario 2

**Table 2:** Estimates of variance components, narrow-sense heritability, dominance ratio, epistatic ratio, broad-sense heritability, heterozygosity effects, and the maximum log-likelihood (Log *L*) for forward prediction scenario 1a/b (1,825 clones from 2013–2015 used as training population)

Trait	Model	${\varvec{\sigma}}_{{\varvec{A}}}^{2}$	${\varvec{\sigma}}_{{\varvec{D}}}^{2}$	${\varvec{\sigma}}_{{\varvec{E}}}^{2}$	${\varvec{\sigma}}_{{\varvec{\varepsilon}}}^{2}$	$\frac{{{\varvec{\sigma}}_{{\varvec{A}}}^{2} }}{{{\varvec{\sigma}}_{{\varvec{P}}}^{2} }}$	$\frac{{{\varvec{\sigma}}_{{\varvec{D}}}^{2} }}{{{\varvec{\sigma}}_{{\varvec{P}}}^{2} }}$	$\frac{{{\varvec{\sigma}}_{{\varvec{E}}}^{2} }}{{{\varvec{\sigma}}_{{\varvec{P}}}^{2} }}$	$\frac{{{\varvec{\sigma}}_{{\varvec{A}}}^{2} + {\varvec{\sigma}}_{{\varvec{D}}}^{2} + {\varvec{\sigma}}_{{\varvec{E}}}^{2} }}{{{\varvec{\sigma}}_{{\varvec{P}}}^{2} }}$	${\varvec{HET}}$	LogLik
TCH	A	98.13 (3.64)			36.09 (0.39)	0.73 (0.008)					− 45,439.59^f^
	AH	95.78 (3.56)			36.09 (0.39)	0.73 (0.008)				125.72 (19.88)	− 45,416.81^e^
	AD	49.91 (5.21)	35.84 (4.70)		36.08 (0.39)	0.41 (0.04)	0.29 (0.04)		0.70 (0.008)		-45,403.70^d^
	ADH	53.41 (5.33)	31.70 (4.63)		36.08 (0.39)	0.44 (0.04)	0.26 (0.04)		0.70 (0.008)	133.89 (29.95)	**− **45,390.63^c^
	ADE	21.91 (4.19)	9.71 (4.19)	44.33 (5.43)	36.06 (0.39)	0.20 (0.04)	0.09 (0.04)	0.40 (0.05)	0.68 (0.009)		**− **45,373.39^b^
	ADEH	23.00 (4.22)	3.13 (3.68)	48.45 (5.30)	36.06 (0.39)	0.21 (0.04)	0.03 (0.03)	0.44 (0.05)	0.67 (0.008)	118.83 (20.66)	**− **45,355.42^a^
CCS	A	0.41 (0.02)			0.134 (0.001)	0.75 (0.007)					6586.014^b^
	AH	0.41 (0.02)			0.134 (0.001)	0.75 (0.007)				0.093 (1.30)	6586.277^b^
	AD	0.40 (0.02)	0.006 (0.014)		0.134 (0.001)	0.74 (0.029)	0.010 (0.026)		0.75 (0.008)		6586.060^b^
	ADH	0.40 (0.02)	0.008 (0.014)		0.134 (0.001)	0.74 (0.03)	0.014 (0.03)		0.75 (0.008)	0.142 (1.36)	6586.363^b^
	ADE	0.256 (0.03)	~ 0 (NA)	0.110 (0.02)	0.134 (0.002)	0.51 (0.05)	~ 0 (NA)	0.22 (0.05)	0.73 (0.008)		6596.317^a^
	ADEH	0.256 (0.03)	~ 0 (NA)	0.11 (0.02)	0.134 (0.002)	0.51 (0.05)	~ 0 (NA)	0.22 (0.05)	0.73 (0.008)	0.204 (1.30)	6596.591^a^
Fibre	A	1.54 (0.05)			0.16 (0.002)	0.91 (0.003)					4118.252^b^
	AH	1.54 (0.05)			0.16 (0.002)	0.91 (0.003)				1.29 (2.47)	4119.29^b^
	AD	1.43 (0.08)	0.08 (0.05)		0.16 (0.002)	0.86 (0.03)	0.05 (0.03)		0.91 (0.003)		4119.76^b^
	ADH	1.43 (0.08)	0.08 (0.05)		0.16 (0.002)	0.86 (0.03)	0.05 (0.03)		0.91 (0.003)	0.921 (2.78)	4120.84^b^
	ADE	1.14 (0.12)	0.01 (0.05)	0.28 (0.10)	0.16 (0.002)	0.72 (0.06)	0.006 (0.03)	0.18 (0.06)	0.91 (0.004)		4123.30^a^
	ADEH	1.14 (0.12)	0.01 (0.05)	0.27 (0.10)	0.16 (0.002)	0.72 (0.06)	0.008 (0.03)	0.17 (0.06)	0.91 (0.004)	0.841 (2.53)	4124.28^a^

$\sigma_{A}^{2}$ = additive genetic variance;$\sigma_{D}^{2}$ = dominance genetic variance; $\sigma_{E}^{2}$ = epistatic (additive–additive) genetic variance; $\sigma_{\varepsilon }^{2}$ = residual variance; $\sigma_{P}^{2}$ = total phenotypic variance; HET = heterozygosity effect. standard error(se) in parantheses.

a–f models without a common superscript are significantly different at *p* < 0.05

Model A = additive model; Model *AH*   additive plus heterozygosity, Model *AD*  additive plus dominance model, Model *ADH*   additive, dominance plus heterozygosity, Model *ADE*   additive, dominance and epistatic effect, Model *ADEH*  additive, dominance, epistatic plus heterozygosity. *TCH* Tonnes of cane per hectare, *CCS*  Commercial cane sugar, measured in percent, Fibre = Fibre content, measured in percent

The estimates of dominance variance were significantly lower for CCS (only explaining up to 6% in scenario 2) and Fibre (up to 5% in scenario1) while using model AD and reduced to almost zero dominance variance when epistatic effects were added in the model AD (Fig. 3). The additive–additive epistatic variance was nearly 30% of the total variance for CCS in both forward prediction scenarios (Fig. 3). The lowest epistatic variance was found for Fibre, which was 19% and 14% in scenarios 1 and 2, respectively (Fig. 3). In general, the proportion of additive variance was reduced when epistatic effects were included in the model for all three traits. Significant dominance variation was only observed for TCH but not for CCS and Fibre.

The above described ADE models were sequentially extended to test if higher-level interactions can explain some phenotypic variation, including additive–dominance and dominance–dominance interaction effects. However, the variance components' estimates associated with those higher-level interaction terms were close to zero for all three traits. Including these interaction effects into the model did not affect the other variance components described above.

### Genome-wide heterozygosity effects

The average heterozygosity across markers was used as a measure of genome-wide heterozygosity per clone. For TCH in scenario 1, the inclusion of heterozygosity in model AD and ADE reduced estimates of dominance variance from (35.84 ± 4.7) to (31.70 ± 4.6) and from (9.71 ± 4.2) to (3.13 ± 3.7), respectively (Table 2). Similarly, in scenario 2, the dominance variance for TCH decreased from (29.59 ± 3.8) to (23.87 ± 3.7) and (11.0 ± 3.5) to (1.24 ± 2.7) when heterozygosity effects were added as a covariate in models AD and ADE, respectively (Table 3). These results suggest that the overall heterozygosity coefficient captures a substantial part of the variation for some traits that would otherwise be ascribed to dominance variance.

**Table 3:** Estimates of variance components, narrow-sense heritability, dominance ratio, epistatic ratio, broad-sense heritability, heterozygosity effects, and the maximum log-likelihood (LogL) for forward prediction scenario 2 (2,397clones from 2013–2016 used as training population)

Trait	Model	$\sigma_{A}^{2}$	$\sigma_{D}^{2}$	$\sigma_{E}^{2}$	$\sigma_{\varepsilon }^{2}$	$\frac{{\sigma_{A}^{2} }}{{\sigma_{P}^{2} }}$	$\frac{{\sigma_{D}^{2} }}{{\sigma_{P}^{2} }}$	$\frac{{\sigma_{E}^{2} }}{{\sigma_{P}^{2} }}$	$\frac{{\sigma_{A}^{2} + \sigma_{D}^{2} + \sigma_{E}^{2} }}{{\sigma_{P}^{2} }}$	${\text{HET}}$	LogLik
TCH	A	85.99 (2.75)			37.39 (0.33)	0.70 (0.007)					− 66,907.76^f^
	AH	83.26 (2.67)			37.39 (0.33)	0.69 (0.007)				139.70 (16.78)	− 66,870.77^e^
	AD	48.84 (4.14)	29.59 (3.80)		37.38 (0.33)	0.42 (0.033)	0.26 (0.032)		0.68 (0.007)		− 66,872.13^d^
	ADH	53.52 (4.25)	23.87 (3.70)		37.38 (0.33)	0.47 (0.03)	0.21 (0.03)		0.67 (0.007)	157.38 (25.40)	− 66,850.68^c^
	ADE	22.82 (3.72)	11.09 (3.49)	38.89 (4.82)	37.37 (0.33)	0.21 (0.033)	0.10 (0.032)	0.35 (0.043)	0.66 (0.007)		− 66,845.05^b^
	ADEH	23.30 (3.67)	1.24 (2.67)	46.47 (4.54)	37.37 (0.33)	0.21 (0.033)	0.011 (0.025)	0.43 (0.04)	0.66 (0.008)	140.57 (17.01)	− 66,813.82^a^
CCS	A	0.367 (0.012)			0.125 (0.001)	0.746 (0.006)					10,890.366^b^
	AH	0.367 (0.012)			0.125 (0.001)	0.746 (0.006)				− 0.801 (1.106)	10,890.728^b^
	AD	0.338 (0.019)	0.021 (0.012)		0.125 (0.001)	0.698 (0.028)	0.044 (0.025)		0.74 (0.007)		10,891.675^b^
	ADH	0.338 (0.019)	0.022 (0.012)		0.125 (0.001)	0.697 (0.028)	0.045 (0.025)		0.74 (0.006)	− 0.764 (1.27)	10,892.093^b^
	ADE	0.228 (0.024)	~ 0 (NA)	0.110 (0.019)	0.125 (0.001)	0.493 (0.045)	~ 0 (NA)	0.237 (0.043)	0.73 (0.007)		10,903.98^a^
	ADEH	0.228 (0.024)	~ 0 (NA)	0.109 (0.019)	0.125 (0.001)	0.49 (0.045)	~ 0 (NA)	0.24 (0.043)	0.73 (0.007)	− 0.811 (1.11)	10,904.35^a^
Fibre	A	1.38 (0.04)			0.16 (0.001)	0.90 (0.002)					6449.904^b^
	AH	1.38 (0.04)			0.16 (0.001)	0.90 (0.002)				0.562 (2.12)	6450.688^b^
	AD	1.31 (0.06)	0.05 (0.04)		0.16 (0.001)	0.86 (0.03)	0.03 (0.03)		0.90 (0.003)		6450.904^b^
	ADH	1.31 (0.06)	0.06 (0.04)		0.16 (0.001)	0.86 (0.03)	0.03 (0.03)		0.90 (0.003)	0.445 (2.34)	6451.769^b^
	ADE	1.13 (0.10)	0.02 (0.04)	0.18 (0.09)	0.16 (0.001)	0.76 (0.06)	0.012 (0.03)	0.123 (0.06)	0.89 (0.003)		6452.861^a^
	ADEH	1.13 (0.10)	0.02 (0.04)	0.18 (0.09)	0.16 (0.001)	0.76 (0.06)	0.014 (0.03)	0.120 (0.06)	0.89 (0.003)	0.263 (2.21)	6453.659^a^

$\sigma_{A}^{2}$ = additive genetic variance;$\sigma_{D}^{2}$ = dominance genetic variance; $\sigma_{E}^{2}$ = epistatic (additive–additive) genetic variance; $\sigma_{\varepsilon }^{2}$ = residual variance; $\sigma_{P}^{2}$ = total phenotypic variance; HET = heterozygosity effect. standard error(se) in parantheses.

a–f models without a common superscript are significantly different at *p* < 0.05

Model A; additive model, Model AH, additive plus heterozygosity; Model AD = additive plus dominance model; Model ADH = additive, dominance plus heterozygosity; Model *ADE* additive, dominance and epistatic effect, Model *ADEH* additive, dominance, epistatic plus heterozygosity. *TCH* Tonnes of cane per hectare, *CCS* Commercial cane sugar, measured in percent; Fibre Fibre content, measured in percent

Our results show that the regression coefficient on heterozygosity was large for TCH (133.89 ± 29.95 and 157.38 ± 25.40 for scenario1 and 2, respectively), suggesting an increase in genome-wide heterozygosity is associated with an average increase in cane yield (Table 2, Table 3). For all traits, estimates of regression coefficients were in a favourable (positive) direction except for CCS in scenario 2 (Table 3). However, the standard error of the regression coefficient for CCS and Fibre was quite large, even larger than for the variance component estimates (Tables 2, 3).

### Model comparison for the goodness of fit

The likelihood ratio test (LRT) criterion was used to compare the nested GBLUP models. The maximum log-likelihood values from all tested models for forward prediction scenarios 1 and 2 are shown in Tables 2 and 3. Based on the LRT criterion, model ADEH was the best fit for TCH, while model ADE was found to fit the data best for CCS and Fibre. It should be noted that there was no significant difference found between models A and AD for CCS and Fibre (Tables 2 and 3). In summary, we found substantial non-additive genetic variance, particularly additive–additive interaction variance for all three analysed traits, while substantial dominance variance was only found for TCH. It is important to note that the methodology we used for constructing the coefficient matrices to partition the genetic variance (the additive, dominance and additive–additive relationship matrices) was developed for diploid organisms. Therefore, the interpretation of these variance components is difficult for sugarcane which has extreme levels of ploidy that vary across the genome.

### Genomic prediction accuracies

**Fig. 4:**
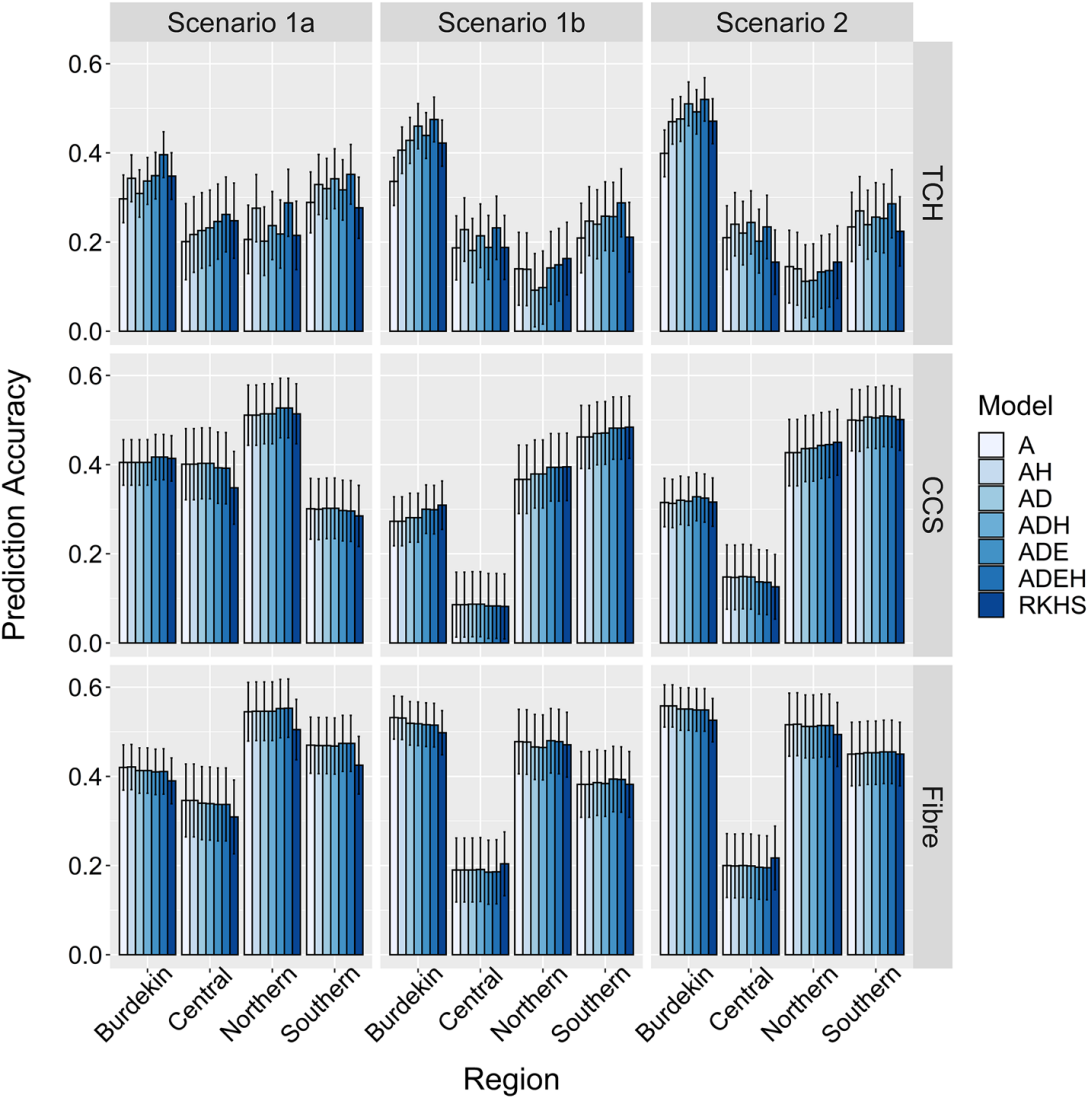
Prediction accuracies for three key traits in different forward prediction scenarios measured as a Pearson’s correlation between genomic prediction and adjusted phenotypes of clones. Prediction accuracies for tonnes cane per hectare (TCH), commercial cane sugar (CCS), and Fibre content in, scenario 1a (1,825 clones from 2013–2015 used as training population to predict 739 clones from 2016); scenario 1b (1,825 clone from 2013–2015 used as training population to predict 691 clones from 2017); and scenario 2 (2,397 clones from 2013–2016 used as training population to predict 691 clones from 2017). Model A = additive model; Model *AH* additive plus heterozygosity; Model *AD* additive plus dominance model; Model *ADH* additive, dominance plus heterozygosity; Model *ADE*  additive, dominance and epistatic effect; Model *ADEH*  additive, dominance, epistatic plus heterozygosity. Error bars show the standard errors of the correlations between the genomic prediction and the adjusted phenotypes

We assessed the predictive ability as the Pearson's correlation between the adjusted phenotypes and the genomic predictions obtained from the compared GBLUP, extended-GBLUP, and RKHS models. As shown in Fig. 4, the accuracy of breeding or clonal performance values differed substantially between traits and regions and through the forward prediction scenarios. Prediction accuracies ranged from 0.297 to 0.409 for CCS and from 0.389 to 0.446 for Fibre in all considered scenarios (Table 4). In contrast, the prediction accuracy was lower (0.218–0.325) for TCH (Table 4). In general, for all traits and irrespective of the model used, the prediction accuracy in scenario 1b (predict 2017 series clones) was consistently lower than scenario 1a (predict 2016 series clones), where both scenarios used the same training population (2013–2015). For TCH, incorporating dominance effects into the prediction model improved the prediction accuracies by approximately 6% and 8% in scenario1a and 1b, respectively, which was further increased by 14 and 18% after using model ADE, compared to the additive model (Table 4).

**Table 4:** Prediction accuracies averaged across four regions of Sugar Research Australia’s breeding program, comparing GBLUP, extended-GBLUP, and RKHS models in forward prediction scenarios 1a (1,825 clones from 2013–2015 used as training population, to predict 739 clones from 2016), scenario 1b (1,825 clones from 2013–2015 used as training population to predict 691 clones from 2017) and scenario 2 (2,397 clones from 2013–2016 used as training population to predict clones from 2017)

	Scenario 1a			Scenario 1b			Scenario 2		
	TS: 2013–2015			TS: 2013–2015			TS: 2013–2016		
	VS: 2016			VS: 2017			VS: 2017		
Model	TCH	CCS	Fibre	TCH	CCS	Fibre	TCH	CCS	Fibre
A	0.248	0.405	0.445	0.218	0.297	0.396	0.247	0.348	0.431
AH	0.291	0.404	0.446	0.255	0.297	0.395	0.28	0.347	0.431
AD	0.264	0.406	0.442	0.235	0.304	0.390	0.262	0.353	0.429
ADH	0.287	0.406	0.442	0.258	0.305	0.390	0.28	0.352	0.429
ADE	0.283	0.409	0.443	0.257	0.315	0.394	0.27	0.354	0.429
ADEH	0.325	0.408	0.444	0.286	0.315	0.393	0.29	0.354	0.428
RKHS	0.272	0.390	0.407	0.246	0.318	0.389	0.247	0.348	0.431

Prediction accuracies were calculated as the Pearson’s correlation between genomic prediction and the adjusted phenotypes

Model A = additive model; Model AH = additive plus heterozygosity; Model AD = additive plus dominance model; Model ADH = additive, dominance plus heterozygosity; Model ADE = additive, dominance and epistatic effect; Model ADEH = additive, dominance, epistatic plus heterozygosity; RKHS = Reproducible kernel Hilbert space. TCH = Tonnes of cane per hectare; CCS = Commercial cane sugar; Fibre = Fibre content. TS = Training data set; VS = Validation data set

Overall, including heterozygosity in the genomic prediction model with non-additive genetic effects led to an increase in prediction accuracy. For TCH, the prediction accuracy obtained from the ADEH model was increased by 31% in scenario 1a and 1b compared to model A (Table 4). For scenario 2, the relative improvement in prediction accuracy in model ADEH compared to model A was 17%. For CCS, including genome-wide heterozygosity did not affect the prediction accuracy, and model ADE performed better than simple additive model (Table 4). The highest average prediction accuracy was observed for Fibre, and it also validates that Fibre has the highest heritability among all three investigated traits. The highest average prediction accuracy for Fibre was 0.445, 0.396, and 0.431 for model A in prediction scenarios 1a, 1b, and scenario 2, respectively (Table 4), while including non-additive genetic effects and heterozygosity did not affect the prediction accuracy.

The RKHS model outperformed the baseline model A for TCH with an average prediction accuracy of 0.272 (compared to 0.248 for model A) and 0. 246 (0.218 for model A) for prediction scenarios 1a and 1b, and 0.251 (0.247 for model A) for scenario 2, respectively (Table 4). In contrast, the extended-GBLUP method with dominance, epistasis, and heterozygosity effects (model ADEH) surpassed the RKHS model for TCH in scenario 1 (1a and 1b) as well as in scenario 2. For CCS, the RKHS model performed better than the additive model in scenario 1b but not in scenario 1a and scenario 2. Regardless of the scenario, for Fibre, the additive model achieved higher prediction accuracies than RKHS (Table 4). In scenario 2, when the training data set was updated by including 2016 series clones, the prediction accuracy was improved compared to scenario 1b (predict 2017 clones from the 2013–2015 training data set) irrespective of the trait. The improvement in prediction accuracy in scenario 2 compared to scenario 1b was 2%, 12%, and 9% for TCH, CCS, and Fibre.

We calculated the standard error of the Pearson correlation *r* which was used as the measure for prediction accuracy as $\sqrt {\frac{{1 - r^{2} }}{n - 2}}$, where *n* is the total number of clones in a particular region (Table 1), and *r* is the correlation between genomic prediction and adjusted phenotypes. For example, the standard error of these correlations was relatively low for the Burdekin region, which reflects that a larger number of clones were tested in this region. Likewise, the highest prediction accuracies were found in the Burdekin region for TCH in all scenarios (Fig. 4). For TCH, regardless of the region, model ADEH outperformed the other models except for the Northern region in scenario 1b and scenario 2, where the lowest prediction accuracy in our study was found (Fig. 4). Across all regions and validation scenarios, the extended-GBLUP models with dominance, epistasis, and genomic-wide heterozygosity effects achieved higher prediction accuracies than the RKHS model (Fig. 4). For both CCS and Fibre, the differences in prediction accuracies between models A and ADE were very low (Fig. 4).

## Discussion

Genomic prediction is a powerful tool for increasing genetic gain in breeding programs (Crossa et al. 2010). However, genomic prediction as a technology has mainly been documented for diploid species and using additive genomic prediction models. For sugarcane, one of the most complex crop species with extreme levels of ploidy there is a substantial gap in the literature on modelling strategies, including the appropriate use of allele dosage information and the partition of the genetic variance into additive and non-additive components. Therefore, we adopted a pseudo-diploid model in our study as a first step towards investigating the relative importance of additive and non-additive genetic components for genomic prediction of complex traits in sugarcane.

### The relevance of additive and non-additive genetic variance for TCH, CCS, and Fibre

In our study, we have attempted for the first time to quantify the amount of non-additive genetic variance for complex commercial traits in a large sugarcane breeding data set using genome-wide SNP markers. Genomic estimated breeding values (GEBVs) or genomic prediction of clonal performance (GPCP) estimates were computed using a two-stage analysis approach based on adjusted phenotypes (BLUPs) which were provided by the commercial breeding program of Sugar Research Australia. The use of BLUPs as response variables can lead to double penalization of the estimated genetic effects but importantly no genomic relationship was fitted to obtain BLUPs in the first stage and the error variances in the trials (final assessment trials, last stage of the breeding program) were reported to be low, due to large plots and high replication numbers across within-region locations. Furthermore, the BLUP approach accommodated small levels of missing data.

For this study, we used genotype data from a recently developed Axiom SNP array which contains over 400,000 SNPs. It was shown that about 40,000 of these SNP were single-dose markers which covered the whole genome (Aitken et al. 2016). In sugarcane, single-dose markers are convenient for genetic and statistical analyses because of their “presence-absence nature” which enables to treat them like diploid markers. This is convenient because the actual ploidy levels of modern sugarcane cultivars are highly variable and have been reported to lie between 6 and 20 (Garcia et al. 2013) and 10–13 (Piperidis and D’Hont 2020). Furthermore, studies have demonstrated that single-dose markers (i.e. markers heterozygous and present on only one copy of the homologous chromosomes) are prevalent throughout the genome (Aitken et al. 2005) and can represent over 75% of polymorphic markers in an individual cross (Baker et al. 2010; George and Aitken 2010).

Generally, it is important to note that the statistical partitioning of genetic variance into additive, dominance, and epistasis components does not allow a biological inference of physiological gene action (Cheverud and Routman 1995; Huang and Mackay 2016). This seems particularly important when highly polyploid species such as sugarcane are considered. For this study, the partitioning of genetic variance was performed using the NOIA model proposed by Vitezica et al. (2017). In the NOIA approach which was developed assuming a diploid model, the genetic variance is decomposed orthogonally by relaxing the assumption on HWE of the population under investigation and assuming linkage equilibrium (LE) among markers. In theory, orthogonal partitioning of variance means that there is no covariance between genetic effects. Introducing new genetic effects into the GBLUP model should therefore not change the estimates of previous variance component estimates, nor the estimates of breeding values and dominance and epistatic deviations (Vitezica et al. 2017).

In our results, for all traits, the estimates of additive variance were reduced when we incorporated non-additive effects into the models, while residual variances remained constant. Therefore, for TCH, h^2^ was reduced significantly from the additive model A to the full model ADE. These findings are in alignment with the sugarcane literature where low narrow-sense heritability estimates for TCH have been reported (Hogarth 1971; Hogarth et al. 1981; Jackson and McRae 2001; Pisaroglo De Carvalho et al. 2014). In contrast, for CCS and Fibre, the estimates of h^2^ and H^2^ did not change significantly when the additive model was extended to incorporate dominance effects. Nevertheless, the variation explained by additive effects declined when we used model ADE for both CCS and Fibre.

The fact that including additional random terms into the model resulted in a further partitioning of previously estimated variance components without affecting the residual variance suggests that the partitioning of additive and non-additive effects are not empirically orthogonal. In fact, strong linkage disequilibrium (LD) among markers in elite sugarcane cultivars might explain some of the confounding results between additive and non-additive genetic effects in this study (Aitken et al. 2006; Raboin et al. 2008). This is particularly important since in our study a pseudo-diploid model was assumed which is unlikely to capture the full genomic and genetic complexity of sugarcane. From the classical quantitative genetics point of view, a confounding effect of LD is quite expected as the (additive) breeding value involves allele substitution effects of genes, which means that, depending on the distribution of allele frequencies, the additive variance component can capture a substantial proportion of non-additive genetic effects in the classical genomic model (Falconer and Mackay 1996). Moreover, this observation was also reported in several empirical and simulation studies (Bouvet et al. 2015; Hunt et al. 2020; Muñoz et al. 2014; Su et al. 2012).

In practice, the assumption of linkage equilibrium (LE) is unlikely to be fulfilled unless only a few sparsely distributed loci are considered across the genome (Jiang and Reif 2015), which is not ideal in terms of exploiting the full potential of genomic prediction. Importantly, the epistatic relationship matrices derived by building Hadamard products of additive and dominant relationship matrices rely on the LE condition (Cockerham 1954; Kempthorne 1954; Vitezica et al. 2017). Consequently, the epistatic variance can only be decomposed into orthogonal additive–additive, additive–dominance, and dominance–dominance interaction variance components under ideal conditions (Vitezica et al. 2017), which are unlikely to be met in the context of a breeding program.

However, Vitezica et al. (2017) recommended the use of the NOIA model to estimate genetic variance components even under the LD assumption, rather than models that assume HWE. Therefore, it is likely that the estimation strategy we used in our study led to some bias in the epistatic variance estimates, although including additive, dominance, and epistasis simultaneously in the genomic prediction model likely reduces biases in estimates for each individual variance component. More research is needed to quantify the extent of the bias in the variance component estimates when LD is strong and populations are not in HWE. Alvarez-Castro and Crujeiras (2019) have recently proposed a theoretical framework for orthogonal decomposition of the total genetic variance under LD. However, the authors point out the complexity associated with genetic patterns due to the presence of LD (Alvarez-Castro and Crujeiras 2019). While the orthogonal decomposition of the genetic variance into additive and non-additive components remains challenging, it needs to be noted that even under strong LD, the performance of the extended GBLUP for genomic prediction is not compromised (Jiang and Reif 2015).

### The effect of heterozygosity on clonal performance

The extended GBLUP models that included dominance and epistatic effects are based on the assumption of a mean of zero for dominance effects. However, this assumption usually fails when directional dominance is present (Falconer and Mackay 1996). Therefore, it was hypothesized in dairy cattle and crossbred pigs that the extended GBLUP model could overestimate the dominance variance when heterozygosity is not explicitly modelled (Aliloo et al. 2017; Iversen et al. 2019). In our sugarcane study, we make the same observation and see a substantial reduction in dominance variance for TCH when genome-wide average heterozygosity per clone is included in addition to a random dominance term in the genomic model. Although the partition of genetic variance suggested substantial dominance variance for TCH, these results have to be treated with caution since a simplified diploid model was used which is unlikely to capture the full complexity of sugarcane. However, our results suggest that there could be elevated levels of non-additive within-loci interaction. The large effect of average heterozygosity we found on TCH suggests that directional dominance might contribute to genetic variation for this trait. This is in alignment with the quantitative genetics theory, which says that large heterozygosity effects can be expected in the presence of directional dominance (Falconer and Mackay 1996).

### Nonparametric approach—reproducible kernel Hilbert space (RKHS) Model

Reproducible kernel Hilbert space (RKHS), a non-parametric regression model, has been advocated as a robust approach to capture epistasis genetic effects in genomic prediction studies (Gianola et al. 2006; Gianola and van Kaam 2008; Jiang and Reif 2015). Most kernels used in RKHS consider the similarity across the individuals within loci, which represents the total genetic value in terms of a weighted sum of additive and dominance effects (Varona et al. 2018). However, the Gaussian kernel that includes the exponential function represents a non-linear transformation of the additive main effects of markers, encoding a class of epistatic genetic effects (Jiang and Reif 2015). Interestingly, in our results, all linear models that included non-additive genetic effects and most linear models that included additive effects only outperformed the RKHS model. Deomano et al. (2020) reported similar genomic prediction accuracies when comparing a similar RKHS model with other additive models in sugarcane. It should be noted that in an RKHS model, the proportion of the genetic variance components is fixed with the bandwidth parameter *h* (Perez and de Los Campos 2014). On the other hand, in extended GBLUP models, as in this study, the proportion of genetic variance depends on the corresponding genetic effects.

### Modelling non-additive genetic effects improve genomic prediction accuracy

By expanding the baseline additive genetic model (model A) to include dominance, epistasis, and average heterozygosity (model ADEH), we saw a significant increase in the prediction accuracy for TCH of at least 17%. Interestingly, although we observed that almost two-thirds of the genetic variance was due to non-additive components for this particular trait, the observed improvement in predictive ability was comparatively small when the model was designed to account for these non-additive effects specifically. Similar results were observed in multiple studies where a significant contribution of non-additive genetic variance was found, but only a slight increment in prediction accuracy was noted (Aliloo et al. 2016; Endelman et al. 2018; Tan et al. 2018). A minor increment in prediction accuracy was observed for CCS when the model was extended to include non-additive genetic effects, whereas the additive model gave the highest prediction accuracy for Fibre. In contrast to TCH, there was no improvement in prediction accuracy for CCS and Fibre when average heterozygosity was added in the GBLUP model. Finally, the prediction accuracy increased marginally regardless of the trait when the training data set was updated by adding clones from the 2016 series (compare scenario 2 vs scenario 1(b). This is in alignment with several reports in the literature that training population must be frequently updated with new phenotyped and genotyped individuals to maintain a high prediction accuracy (e.g. Auinger et al. 2016; Podlich et al. 2004).

In our study, we validated genomic prediction accuracies across years in three forward prediction scenarios using two years to assess the level of variation in prediction accuracies that could be expected in a commercial breeding context, as well as to investigate the effect of training population size on the prediction accuracies for all three traits. Furthermore, we observed a high level of variation in prediction accuracies between the four different regions. In the Australian sugarcane industry, separate breeding regions were developed to account for substantial climatic and environmental differences between different production regions. Within Australia, the importance of genotype-environment interactions and its impact on selection was reported in different sugarcane studies (Bull et al. 1992; Jackson et al. 1995; Jackson and Hogarth 1992; Mirzawan et al. 1994). These studies indicated that genotype-crop year interaction was small compared to the genotype-location interaction regardless of the region where these studies were conducted. Our observed variation in prediction accuracy among the regions might be due to between-region variation.

Interestingly, we observed a decline in the prediction accuracy for all three traits in scenario 2 (model was trained with 2013–16 to predict 2017 clones) compared to scenario 1a (model was trained with 2013–15 to predict 2016 clones). One possible reason might be the genetic relatedness between the validation population and the training population was smaller compared to forward prediction scenario 1a. However, comparing the average top 10% genomic relationships between the reference and the validation population, which was shown to give a robust estimate of the genetic connectedness between the training and validation population (Daetwyler et al. 2012) showed very similar average relationship estimates of 0.142 and 0.146 for scenarios 1a and 2, respectively. Thus, the decline in prediction accuracy in scenario 2 compared to scenario 1a could rather be attributed to large year effects and genotype by year interaction, rather than differences in genetic relationships between training and validation populations.

A particular challenge in sugarcane is the high level of ploidy that has been shown to be a cause of phenotypic variation through substantial allele-dosage effects on genic expression (Osborn et al. 2003; Soltis et al. 2016). Recent work has investigated the effect of allele-dosage on genomic prediction accuracy in autotetraploid species, such as potatoes and blueberries, using pseudo-diploid, tetraploid, and continuous parametrization approaches (Amadeu et al. 2020; Endelman et al. 2018; Oliveira et al. 2018). Interestingly, Amadeu et al. (2020) reported no difference in terms of prediction accuracy between models considering additive and non-additive genetic components that were built using tetraploid and diploid parameterization approaches. For sugarcane, however, the implementation of allele dosage in GS is much more complicated due to varying levels of ploidy across the genome (Garcia et al. 2013; Piperidis and D’Hont 2020). Using currently available sugarcane genotyping technologies, it is a big challenge to categorize heterozygotes correctly when loci have more than two alleles. Due to the complexity involved in defining the allelic dosage, single dosage markers are often used in linkage analyses for polyploidy crops because of the diploid segregation pattern (Aitken et al. 2005; Wu et al. 1992). Therefore, it is likely that a fraction of phenotypic variation that arises from allelic dosages was not accounted for in this study. Further improvements in prediction accuracy of breeding and clonal performance could be achieved if allele dosage was appropriately accounted for. However, it can be assumed that for accurate estimation of additive and non-additive genetic variance in the highly polyploid and heterozygous species sugarcane, the data should be substantial in its size compared to other major crops with comparatively simpler genomes.

## Conclusion

Overall, our results imply that non-additive effects are an important source of phenotypic variation for TCH, CCS, and Fibre in sugarcane. Using a simplified diploid model that was used as a basis to model additive and non-additive components, significant additive–additive epistatic interaction effects were found for TCH, CCS, and Fibre and dominance effects were observed for TCH but not for CCS and Fibre. It is important to note that the biological interpretation of genetic variance decomposition is extremely difficult, even in diploid species, and that the genetic trait architecture cannot be inferred based on statistical variance partitioning. For the highly complex crop species sugarcane, it is likely to be even more difficult to infer the underlying gene action (allelic or non-allelic interactions), especially without specifically accounting for correct allele dosages. Nevertheless, using a pseudo-diploid approach to model additive and non-additive components in a GBLUP framework significantly improved prediction accuracies for key commercial traits. Interestingly, for TCH we also found significant effects of average heterozygosity, which suggests that directional dominance could play an important role for this trait. Including average heterozygosity as a covariate in the model could further improve the prediction ability. Notably, the extended GBLUP models we used in our study consistently outperformed the non-parametric RKHS approach. Therefore, the extended GBLUP approach using a pseudo-diploid parameterization appears to be a convenient and robust way for improving clonal prediction in sugarcane while doubtlessly further research is required to better capture the complexity arising from allele dosage. A more meaningful interpretation of the genetic variance components requires further research focused on developing polyploid modelling approaches for sugarcane as it has already been done for autopolyploid crops with comparatively simpler genomes. Based on the results from our study, we suggest that non-additive effects should be considered in the design of crossing schemes to exploit specific combining ability and maximize offspring performance, especially for highly complex traits such as TCH. The results from our study and previous studies are encouraging that genomic selection is a promising breeding tool that could help to increase genetic gain in sugarcane breeding. Future research focused on both empirical validation and simulation will improve our understanding of how the extreme genomic complexity of sugarcane could be better captured in genomic selection approaches beyond pseudo-diploid parameterization approaches, and how the technology could most efficiently be integrated into commercial breeding programs.
